# Early diagnosis of infantile-onset lysosomal acid lipase deficiency in the advent of available enzyme replacement therapy

**DOI:** 10.1186/s13023-019-1129-y

**Published:** 2019-08-14

**Authors:** Jennifer L. Cohen, Jessica Burfield, Karen Valdez-Gonzalez, Angela Samuels, Arianna K. Stefanatos, Marc Yudkoff, Helio Pedro, Can Ficicioglu

**Affiliations:** 1 Division of Human Genetics, Department of Pediatrics, The Children’s Hospital of Philadelphia, 3401 Civic Center Blvd, Philadelphia, PA 19104 USA; 2 Perelman School of Medicine, University of Pennsylvania, 3400 Civic Center Blvd, Philadelphia, PA 19104 USA; 3 Hackensack University Medical Center, 30 Prospect Ave, Hackensack, NJ 07601 USA; 4 Division of Metabolism (Biochemical Genetics), The Children’s Hospital of Philadelphia, 3501 Civic center Blvd #9054, Philadelphia, PA 19104 USA

**Keywords:** Wolman disease, Enzyme replacement therapy, Lysosomal acid lipase deficiency, Diet, Adrenal insufficiency

## Abstract

**Background:**

Lysosomal acid lipase deficiency (LAL-D) is an autosomal recessive disorder that can present as a severe, infantile form also known as Wolman disease. We sought to determine the outcomes and clinical needs of infants diagnosed with LAL-D, treated with enzyme replacement therapy (ERT).

**Methods:**

A chart review was conducted on two infantile-onset LAL-D patients to determine clinical outcomes based on laboratory results, abdominal imaging, growth and dietary records, cardiology, endocrinology, ophthalmology, hematology, and neurocognitive evaluations.

**Results:**

Two patients, both diagnosed and treated before 6 months old, demonstrated clinical improvement following weekly ERT. They required dosage increases to optimize growth and symptomatology. Both received a formula low in long chain triglycerides and high in medium chain triglycerides, an intervention that allowed significant catch-up growth. Patient 1 required treatment for partial adrenal insufficiency and hypothyroidism. Both patients demonstrated reduction in liver and spleen size and varying degrees of improved liver function. Neither experienced serious adverse reactions to ERT.

**Conclusion:**

ERT has led to longer and healthier survival of affected infants. It is imperative that dietary interventions and systemic clinical care become integral to the management. Continued evidence of survival and clinical improvement in this population, coupled with available mass spectrometry enzyme assay from dried blood spots, raises the question of this rare and possibly underdiagnosed disorder’s candidacy for newborn screening.

**Electronic supplementary material:**

The online version of this article (10.1186/s13023-019-1129-y) contains supplementary material, which is available to authorized users.

## Background

Lysosomal acid lipase deficiency (LAL-D) is an autosomal recessive disorder that can present as a severe, infantile form also referred to as Wolman disease (OMIM #278000) [1]. Prior to the advent of enzyme replacement therapy (ERT) with a recombinant form of lipoprotein lipase, sebelipase alfa (Kanuma®), life expectancy was typically less than twelve months [2]. The only available treatment options (liver transplantation and hematopoietic stem cell transplant) were rarely successful in extending survival and were fraught with additional medical complications [3–6]. Approval of sebelipase alfa (Kanuma®) in 2015 by both the Food and Drug Administration (FDA) and the European Medicine’s Agency (EMA) has enabled longer survival and better quality-of-life, as demonstrated by prior clinical studies [7, 8]. The recommended starting dosage according to the prescribing information is 1 mg/kg administered weekly via intravenous infusion. The product label also states that for patients without optimal clinical response, the dosage should be increased to 3 mg/kg weekly. Lastly, the manufacturers note that in the 9-patient infant clinical trial with rapidly progressive LAL-D, a patient was increased to 5 mg/kg weekly at week 88 of treatment [9].

Infants treated with ERT enjoy improved hepatic function, somatic growth, and amelioration of gastrointestinal and hematologic complications [9]. As patients now survive beyond infancy, it is necessary to consider additional manifestations of disease and to respond with appropriate interventions. For instance, prior studies have reported a low-fat diet as a supportive and/or complementary treatment [9]. Other data underscore a need to be attentive to adrenal complications that may occur in utero [10, 11].

We report two patients who presented at two and six months of age, respectively. Both patients presented with failure to thrive and gastrointestinal issues (vomiting and diarrhea). Imaging studies and laboratory data pointed to a diagnosis of infantile-onset LAL-D. The diagnostic suspicions in these two cases were then confirmed with both enzyme assay and molecular genetic analysis. The clinical course of the two infants was somewhat different, but both cases highlight the need for early diagnosis and swift initiation of ERT as well as the importance of detecting and treating disease manifestations that may not be immediately corrected by ERT.

## Results

### Clinical manifestations and laboratory evaluations

Table 1 depicts the two patients’ similarities and differences in clinical course and progression of growth and development. Both patients demonstrated absent lysosomal acid lipase enzyme activity as well as confirmed pathogenic variants in *LIPA* with targeted gene sequencing. Their parallel presentations of failure to thrive secondary to feeding difficulties and gastrointestinal symptoms, coupled with hepatomegaly and adrenal calcifications, are consistent with the classic presentation of this disease [12].

**Table 1:** Clinical information for two patients with infantile-onset lysosomal acid lipase deficiency treated with enzyme replacement therapy (ERT)

	Patient 1	Patient 2
Current age	27 months	22 months
Sex	Male	Female
Molecular results from *LIPA* gene sequencing	Compound heterozygous pathogenic variants: c.539-2A>G and c.684delT	Homozygous for c.656 T>G (p.Leu219*)
Lysosomal acid lipase enzymatic result at diagnosis	0.0 nmol/h/mL (Normal: > 21.0)	0.0 nmol/h/mL (Normal: > 21.0)
Age at diagnosis	6.5 months	2 months (1 month corrected age; born at 36-weeks gestation)
Age at initiation of ERT	7 months	2.5 months
Presentation at diagnosis	Failure to thrive, hepatosplenomegaly, poor feeding, blood in stool, constipation, spitting up	Failure to thrive, hepatosplenomegaly, loose stools, no vomiting, anemia and thrombocytopenia
Adrenal calcifications present at diagnosis	Yes	Yes
Weight Z scores:		
at diagnosis	−3.52	−3.19 (−1.48 for corrected age)
after 8 months of ERT	−2.48	−3.27 (− 3.04 for corrected age)
after 18 months of ERT	−1.7	− 1.18 (− 1.03 for corrected age)
Adrenal insufficiency	Partial	None
Other endocrinology concerns	Hypothyroidism	
Hematologic concerns	Mild and stable anemia, now resolved; intermittent eosinophilia, now resolved	Secondary hemophagocytic lymphohistiocytosis; iron deficiency anemia
Cardiac evaluation	Unremarkable	Innocent murmur
Developmental Milestones	Receives early intervention	Receives early intervention
Sitting	7 months	11 months
Walking	13.5 months	19 months
Speech	Mildly delayed	Couple of words at 20 months
Current diet	Monogen formula, with fat restriction from foods, and additional medium-chain triglyceride (MCT) oil	Monogen 24 kcals/oz. ad lib (~ 20 oz. per day), with regular diet
ERT side effects	Elevated temperature for one occurrence	Urticaria
ERT dosage	First dose 1 mg/kg; after 3 weeks increased to 3 mg/kg; after 17 months of ERT, increased to 5 mg/kg	Increased from 3 mg/kg to 5 mg/kg

The progression of laboratory studies in response to ERT is depicted in Table 2. In Patient 1, treatment consistently reduced aspartate aminotransferase (AST) levels and maintained stable values for most other parameters. After 22 months of ERT, Patient 1 experienced a 59% reduction in AST (currently within normal limits) and a 32% reduction in ALT (currently within normal limits); the total bilirubin and alkaline phosphatase were within normal limits prior to ERT. Oxysterol levels on plasma analysis in this infant were elevated but down-trending after 12 months of ERT; after 22 months of ERT, the cholestane-3beta,5alpha,6beta-triol on blood spot analysis was within normal limits based on the blood spot reference range of normal. Prior to ERT, Patient 1’s liver volume was 338 mL (weight-adjusted normal 144 mL) and his splenic volume was 35 mL (weight-adjusted normal 11.53 mL). After 12 months of ERT, his liver measured 389 mL (weight-adjusted normal 247.5 mL) and his spleen was 43 mL (weight-adjusted normal 19.8 mL). Thus, hepatic volume declined from 2.3X to 1.6X normal and splenic volume from 3X to 2X normal. Prior to ERT, ultrasound demonstrated normal echogenicity of liver and spleen without focal lesions. The MRI after 12 months of ERT showed normal signal and no focal lesions of the liver or spleen.

**Table 2:** Laboratory and imaging results for two patients with infantile-onset lysosomal acid lipase deficiency treated with enzyme replacement therapy (ERT)

	Laboratory/imaging results in relation to ERT	Albumin (g/dl) (normal range for age)/SI units (g/L)	AST (U/L) (normal range for age)/SI units (μkat/L)	ALT (U/L) (normal range for age)/SI units (μkat/L)	GGTP (IU/L) (normal range for age)/SI units (μkat/L)	Ferritin (ng/ml) (normal range for age)/SI units (pmol/L)	PT (secs) (normal range)	PTT (secs) (normal range)	INR	Total cholesterol (mg/dL) (normal range for age)/SI units (mmol/L)	TG (mg/dL) (normal range for age)/SI units (mmol/L)	LDL (mg/dL) (normal range for age)/SI units (mmol/L)	HDL (mg/dL) (normal range for age)/SI units (mmol/L)	Liver size (cm)	Spleen volume (ml)
Patient #1	prior to ERT	4.4 (3.1–4.2)/44	141 (20–64)/2.35	59 (12–42)/0.99	22/0.37	45.1 (10–112)/101.34	13.5 (11.6–13.8)	28.3 (22–36)	1.08	173 (45–182)/4.48	190 (27–125)/2.15	117 (63–129)/3.03	18 (35–82)/0.47	11.9^*b*^	35^*b*^
	after 3 months ERT	4.4 (3.3–4.3)/44	107 (20–64)/1.79	36 (12–42)/0.60		26.5 (10–112)/59.55	13.5 (11.6–13.8)	29.2 (22–36)	1.08	185 (45–182)/4.79	95 (27–125)/1.07	141 (63–129)/3.65	25 (35–82)/0.65		
	after 12 months ERT	3.6 (3.5–4.6)/36	81 (20–60)/1.35	48 (5–45)/0.80		19.4 (10–70)/43.59	13.9 (11.6–13.8)		1.11	238 (45–182)/6.16	129 (27–125)/1.46	191 (63–129)/4.95	21 (35–82)/0.54	12.4^*b*^	43^*b*^
	after 18 months ERT	3.8 (3.5–4.6)/38	91 (20–60)/1.52	56 (5–45)/0.94		15.5 (10–70)/34.83	11.8 (10.9–13.4)	30.1^*a*^ (22–36)	0.97	141^*a*^ (45–182)/3.65	221^*a*^ (27–125)/2.50	86^*a*^ (63–129)/2.23	11^*a*^ (35–82)/0.28		
	after 22 months ERT	4.0 (3.5–4.6)/40	58 (20–60)/0.97	40 (5–45)/0.67		13.5 (10–99.9)/30.33				128 (45–182)/3.32	99 (27–125)/1.12	89 (63–129)/2.31	19 (35–82)/0.49		
Patient #2	prior to ERT	2.5 (3.4–4.2)/25	189 (0–120)/3.16	62 (0–28)/1.04	453 (6–19)/7.57	7640 (9–120)/17,167.08	13.6	25.2	1.04	224 (0–200)/5.80	593 (60–160)/6.70	TG result too high for accurate LDL estimation; LDL direct: 95 (0–159)/2.46	34 (35–150)/0.88	10^*c*^	157^*c*^
	after 3 months ERT	3.7 (3.4–4.2)/37	61 (20–60)/1.02	54 (5–45)/0.90	290 (6–19)/4.84	39.90 (9–120)/89.66	14.4	33.9	1.11	167 (0–200)/4.33	114 (60–160)/1.29	126 (0–129)/3.26	18 (35–150)/0.47		
	after 12 months ERT	3.9 (3.4–4.2)/39	55 (20–60)/0.92	58 (5–45)/0.97	97 (6–19)/1.62	21.8 (9–120)/48.98	14.4 (12.2–14.4)	27.1	1.11	191 (0–200)/4.95	159 (60–160)/1.80	139 (0–129)/3.60	20 (35–150)/0.52	8.6^*c*^	127^*c*^

*AST* Aspartate aminotransferase, *ALT* Alanine aminotransferase, *GGTP* Gamma glutamyl transpeptidase, *PT* Prothrombin time, *PTT* Partial thromboplastin time, *TG* Triglycerides, *LDL* Low-density lipoprotein, *HDL* High-density lipoprotein,^*a*^After 15 months of ERT, ^*b*^based on MRI, ^*c*^based on ultrasound

ERT in Patient 2 either normalized or markedly reduced elevated levels of triglycerides, low-density lipoprotein, ferritin, and AST. Patient 2 also demonstrated normalization of pre-treatment hypoalbuminemia and improvement in an elevated blood level of γ-glutamyl transpeptidase (GGTP). After 12 months of ERT, Patient 2 experienced an appropriate increase in albumin (56%) to a level within normal range, and a 71% reduction in AST (currently within normal limits), a minimal 6% reduction in ALT (still mildly elevated), and a 79% reduction in GGTP (though still elevated). Similar to Patient 1, total bilirubin and alkaline phosphatase were within normal limits prior to ERT. Abdominal ultrasound imaging in Patient 2 confirmed a reduction in the size of both her liver and spleen. Prior to ERT, her liver measured 10.1 cm (age-adjusted normal 4-9 cm) and her spleen was 9.7 cm (age and sex-adjusted normal 3.2–5.5 cm). After 12 months of ERT, her liver went from 1.1X the upper limit of normal to within normal range at 8.6 cm (age-adjusted normal 6.5–10.5 cm) and her spleen went from 1.8X the upper limit of normal to 1.2X the upper limit of normal at 9.6 cm (age and sex-adjusted normal 5.1–8.2 cm) [13, 14]. Prior to ERT, ultrasound demonstrated normal echogenicity of liver and spleen without focal lesions. After 18 months of ERT, Patient 2 demonstrated parenchymal heterogeneity of the liver, which was essentially unchanged from prior studies, and the echogenicity of the spleen was homogenous without focal lesion.

### Enzyme replacement therapy dosage and adverse reactions

Patient 1 received 1 mg/kg/dose for the initial 3 weeks, but this was increased to 3 mg/kg/dose due to inadequate weight gain. Dosage was increased after 17 months of therapy to 5 mg/kg/dose because of abdominal bloating and distension and subsequent feeding difficulties even with adequate weight gain. Patient 2 initially received 3 mg/kg/dose and then was increased to 5 mg/kg/dose because of persistently abnormal laboratory results. Based on the clinical trial conducted in infant patients [9], in which two patients were increased to a dose of 5 mg/kg, which is also reported on the package insert for Kanuma®, both patients reported here were similarly increased to the highest reported dosage, 5 mg/kg, to achieve optimal clinical improvement. Only Patient 1 in our cohort was tested for the presence of drug antibodies at the time of this manuscript, and was found to be negative for antibodies after 18 months of weekly ERT.

A single episode of higher core temperature (37.8–37.9 degrees Celsius) occurred during ERT infusion in Patient 1. This resolved spontaneously. Patient 2 developed urticaria with ERT and the reaction of urticaria continued for 5 weeks of treatment. At the time of the initial reaction, she was treated with an antihistamine, corticosteroids and an antipyretic. The patient was then started on a combination of corticosteroid and H1 antihistamine for 4 weeks due to the severity of the urticaria, followed by an H1 antihistamine continued for an additional 4 weeks, after which time, all pre-infusion medications were discontinued, and no other adverse events occurred. She is now receiving home infusions following one year of hospital-based infusions, and is not requiring any pre-medications.

### Growth and dietary management

Patients 1 and 2 both demonstrated catch-up growth following initiation of ERT coupled with introduction of a metabolic formula low in long chain triglycerides (LCT) and high in medium chain triglycerides (MCT).

Due to vomiting and bloody stool early in infancy, Patient 1 had been switched from breast milk to Nutramigen formula, and then to Alfamino by 2 months of age. At approximately 5 months old, his weight had precipitously fallen below his age group’s growth curve, and at the time of diagnosis (~ 6 months old) the weight and height were respectively at Z-scores of −3.50 and −1.81. After confirmation of the diagnosis, Patient 1 was slowly transitioned (over two weeks) to Monogen (24 kcal/30 mL). He continued to receive pureed baby foods and cereal. During the month following his transition to this formula, he was gaining an average of 22 g/day (expected weight gain for his 8 month old age: 9 g/day). Approximately 1 month after the diagnosis was confirmed, 5 mL/day of MCT oil was also added to his nutritional regimen. One week following this addition, he was gaining 35 g/day, demonstrating excellent catch-up growth. Subjectively, his parents noted greater satiety with the new formula, but also reported increased constipation.

The rate of weight gain in Patient 1 declined to 5.7 g/day by 2–3 months after the start of dietary intervention, thus prompting an increase in dietary LCT (as whole fat baby yogurt) from 4 to 16% of total fat. Weight gain then increased within 1 week to 16.4 g/day. After six months of ERT (age 12 months) caloric density was increased to 27 kcal/30 mL formula and his Z-scores for weight and height improved respectively to −2.26 and −1.89. By age 14 months, most fat (80% of calories) was provided as MCT and the remainder from LCT. After a full year of diet therapy and ERT (age 18 months) he gained an average of 5 g/day (normal 4–10 g/day) and his Z-score for weight (−1.93) and height (−1.55) continued to improve. The growth velocity was 2.6 cm/month, exceeding the expectation for age. After eighteen months on ERT (age 24 months) his nutrition goals were 120-125 kcal/kg of which 30% came from fat (80% MCT, 20% LCT). Throughout his life, his head circumference consistently showed a Z-score of −1.12. Due to the LCT-restricted diet and risk for fat-soluble vitamin deficiencies, Patient 1 was evaluated and demonstrated normal/high levels of vitamin D and vitamin E and therefore no supplementation was required.

At the time of Patient 2’s diagnosis (age 2 months, corrected age 1 month based on her preterm 36-week gestation), she was switched from Enfamil 24 kcals/30 mL to Monogen 24 kcal/30 mL. At 20 months old, she received ad lib Monogen (~ 600 mL/day); LCT content from her solid food intake was not restricted. Her weight percentile increased from <5th percentile at diagnosis (6.94% for corrected age, Z = −1.48) to just below the 10th percentile by 19.5 months old (15.13% for corrected age, Z = −1.03). Her weight-for-length percentile improved overall as well, moving from 12th percentile (Z = −1.18) to 5th percentile (8 months old), but most recently, to 50th percentile (20 months old).

### Clinical manifestations outside the therapeutic scope of ERT

Patient 1 showed bilateral adrenal calcifications during the initial diagnostic evaluation, a finding that was likely present prenatally based on maternal report of fetal ultrasound findings. A poor cortisol response to ACTH stimulation pointed to partial adrenal insufficiency, which was controlled with stress dose steroids rather than maintenance therapy. Hypothyroidism was manifested by a persistent increase of thyroid stimulating hormone (TSH) first noted at 11 months old (with subsequently normal total and free thyroxine levels); his thyroid peroxidase antibodies were mildly positive. Of note, his TSH level had been previously normal at 6 months of age during his initial diagnostic evaluation for failure to thrive. He is currently treated with daily levothyroxine therapy, in accordance with treatment practice for an infant with TSH persistently greater than 10. Patient 2 was twice evaluated for adrenal insufficiency but showed no evidence of this endocrinopathy. Additionally, her TSH level was evaluated once at the time of her diagnosis (age 2 months) and was within the range of normal for age.

Patient 1 has had intermittent mild eosinophilia with two separate episodes of an elevated absolute eosinophil count. With treatment of hypothyroidism, the eosinophilia remitted. Patient 1 also presented with a mild and stable anemia (hemoglobin 10.1 g/dL) but a normal reticulocyte count and a peripheral blood smear without suggestion of hemolysis. Eventually, his complete blood count (CBC) normalized. Patient 2 presented with severe anemia (hemoglobin 6.8 g/dL) and thrombocytopenia (platelet count 50 K/uL). She was diagnosed with secondary hemophagocytic lymphohistiocytosis, which has been previously noted in infantile LAL-D patients [15]. Her anemia was determined to be secondary to both the hematologic-related complications of infantile LAL-D, as well as iron deficiency*.* Shortly after initiation with ERT, her hemoglobin level rose to 9.6 g/dL, and platelet count to 116 K/uL. After 18 months of ERT, her hemoglobin was stable at 9.2 g/dL and her platelet count continued to improve to 178 K/uL.

Both patients received cardiac evaluations. After 12 months of ERT, Patient 1 had a normal cardiology-focused physical examination, electrocardiogram and echocardiogram. A repeat cardiac evaluation (at 18 months) was unchanged. A similar cardiac evaluation was normal for Patient 2. A celiac panel sent at age 24 months on Patient 1 showed slightly elevated anti-tissue transglutaminase IgA but negative anti-endomysial IgA antibodies and normal immunoglobulin A. The celiac panel had been normal at the time of initial diagnosis (6 months old); poor weight gain, however, prompted the repeat test. A general ophthalmologic examination in Patient 1 was normal. A more specialized exam and imaging completed by an ophthalmic geneticist determined on slit lamp examination that the patient had an entirely normal cornea and anterior segment; the patient’s cornea was clear without accumulation, and his fundoscopy exam was also normal. Functional studies of rods and cones (electroretinogram) were not performed because extensive ophthalmic imaging (blue light autofluorescence imaging, optical coherence tomography, and fundoscopic pictures) demonstrated an entirely normal retinal phenotype.

### Neurocognitive outcomes

Patient 1’s early developmental trajectory was unremarkable according to parent report, with milestones such as first words and steps emerging within the expected time frames. He participated in feeding therapy from 12 to 18 months of age and has had developmental instruction through Early Intervention beginning at 8 months of age. At 29 months of age, a neurodevelopmental evaluation using the Bayley Scales of Infant Development, Third Edition (Bayley-III) indicated that his overall cognitive development was in the High Average range (scaled score (ss) = 13) when compared to same-age peers. Receptive language skills were in the Superior range (ss = 15) and his expressive language skills were in the High Average range (ss = 13). Fine and gross motor skills were considered Average for his age (ss = 10 and 8, respectively). In order to document his level of function in everyday interactions and activities, a caregiver completed the Vineland Adaptive Behavior Scales, Comprehensive Report Form, Third Edition (Vineland-3). On this measure, the patient’s communication (receptive and expressive), socialization, and motor (gross and fine) skills were estimated to be within the Average range (Standard Score (SS) = 104, 92, and 104, respectively), while estimates of his daily living skills (e.g., dressing, feeding, etc.) were in the Low Average range for his age (SS = 85). Parent responses on the Achenbach Child Behavior Checklist (CBCL) revealed no significant concerns regarding emotional or behavioral functioning.

For Patient 2, her development has been progressing as follows: At 18 months of age she was cruising, and was walking at 19 months old, which demonstrates mild gross motor delay. She has been age-appropriate for her receptive language skills but has had delayed expressive language skills with only a couple of words at 20 months old. She has thus far demonstrated age-appropriate social development and she is receiving early intervention services. A formal neurocognitive evaluation is pending at the time of this manuscript.

## Discussion

Prior clinical trials document the safety and efficacy of sebelipase alfa to treat infantile-onset LAL-D. The two patients presented here are further examples of this treatment’s safety (no serious adverse events) and efficacy (improvement in clinical symptoms, laboratory parameters, survival, and growth). Therapeutic efficacy is also reflected by a reduction of hepatosplenomegaly in both patients as well as symptomatic improvement in gastrointestinal function.

Relatively scant attention has been devoted to dietary management for infantile LAL-D. Our two patients showed improved growth during ERT when they received a specialized formula either with (Patient 1) or without (Patient 2) an additional fat-restricted diet. Of note, Patient 1 who has been on a fat-restricted diet showed a greater growth improvement (based on Z-scores) compared to Patient 2 (Additional file 1: Figure S1). Regular dietary adjustments appear to be indicated in order to optimize growth. It is important to continuously evaluate the patient’s intake to ensure fat consumption remains within the recommended goals. Based on the use of low-fat diet management in late-onset LAL-D to help treat patients’ dyslipidemia and hepatic impairment [16] we speculate that an LCT-restricted and MCT-enriched diet is beneficial in infantile LAL-D for the following reasons: the accumulation of fat in the cells of the small intestine causes swelling and flattening of the intestinal villi, resulting in impaired absorptive ability. Treatment with a low LCT diet reduces further fat accumulation in the intestines and liver. MCT is provided to these patients because it enters the liver directly via the hepatic portal vein and is then metabolized by beta oxidation. MCT is more effectively absorbed and therefore a smaller amount is re-esterified or elongated to LCTs. There is a risk of fat-soluble vitamin deficiencies with this diet, therefore vitamin levels should be evaluated, but only supplemented if there is evidence of deficiency, given the risk of toxicity with administration of fat-soluble vitamin supplements.

A limitation of the study is the small sample size of patients, and therefore, further research is required to study the utility of LCT restriction in this patient population. An additional limitation of the study is that although more advanced imaging techniques are being used presently to assess liver fibrosis and fat content, these were not available methodologies at the time of diagnosis of these cases. Furthermore, there was no clinical indication for liver biopsies in either Patient 1 or 2, given the invasive nature of this test and the age of the patients; therefore we are not aware of the potential degree of liver fibrosis. Lastly, due to the recent advent of available ERT for infantile LAL-D, our clinical results are short-term outcome data, and longer follow-up is required to determine continued clinical management of this patient population.

## Conclusions

With the advent of ERT, extra-hepatic manifestations of infantile-onset LAL-D, including adrenal calcifications and anemia, demonstrate the need for comprehensive clinical management, including thorough endocrinology and hematology evaluations, in order to detect treatable manifestations of disease which are not remediated by ERT. Furthermore, in the era of expanding newborn screening, it is equally important to consider whether this rare but life-threatening disease may benefit from rapid and earlier diagnosis. The present ability to accurately measure lysosomal acid lipase enzyme level from a blood spot using a specific substrate and UPLC-MS/MS [17], potentially further qualifies this disease as a future candidate for newborn screening.

## Methods

Chart review was conducted on two infantile-onset LAL-D patients with special reference to overall clinical course, dietary intake and nutritional recommendations, physical growth, laboratory testing, and imaging results. Patient 1 was previously published as a case report [18]. Informed consent was obtained from both patients’ parents to use their medical information for the purposes of this study.

Both patients received ERT immediately following diagnosis and continued on weekly infusions for at least 20 months. Management in both cases comprised a team of subspecialists, including biochemical geneticists and metabolic dietitians. Lysosomal acid lipase enzyme deficiency and blood oxysterol levels (Patient 1) were measured in Mayo Clinic Laboratory. All other laboratory tests were performed at the patients’ respective treating institutions, Children’s Hospital of Philadelphia (Patient 1) and Hackensack University Medical Center (Patient 2).

## Additional file

Additional file 1: **Figure S1A.** Growth charts for Patient 1. **Figure S1B**. Growth charts for Patient 2. (PDF 2720 kb)

## Data Availability

All data generated or analyzed during this study are included in this published article (and its supplementary information files).

## Abbreviations

AST: aspartate aminotransferase
CBC: complete blood count
ERT: Enzyme replacement therapy
LAL-D: Lysosomal acid lipase deficiency
LCT: long chain triglycerides
MCT: medium chain triglycerides
SS: standard score
TSH: thyroid stimulating hormone
