# Supplementary material for: Early diagnosis of infantile-onset lysosomal acid lipase deficiency in the advent of available enzyme replacement therapy
Source: Orphanet J Rare Dis. 2019 Aug 14;14:198. doi: 10.1186/s13023-019-1129-y (PMC6692931; doi:10.1186/s13023-019-1129-y)
Supplement: Supplementary file 1 — Figure S1A. Growth charts for Patient 1. Figure S1B. Growth charts for Patient 2. (PDF 2720 kb) [file 13023_2019_1129_MOESM1_ESM.pdf]

Weight for age percentiles (Boys, birth to 36 months)

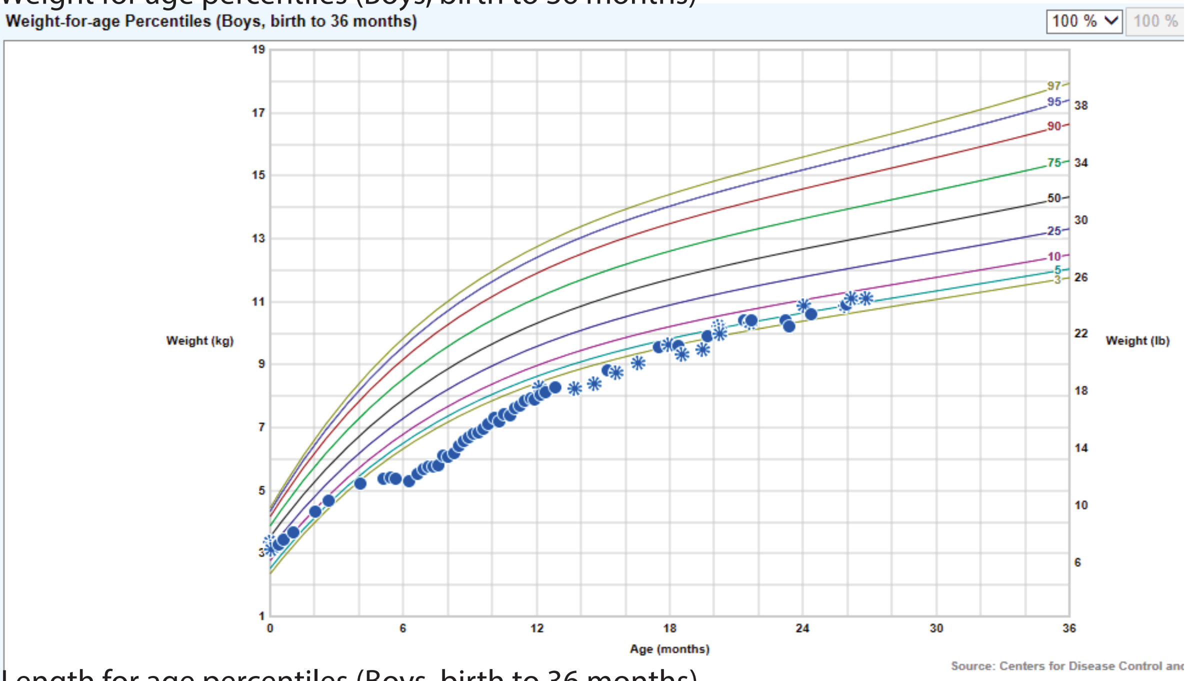

Length for age percentiles (Boys, birth to 36 months)

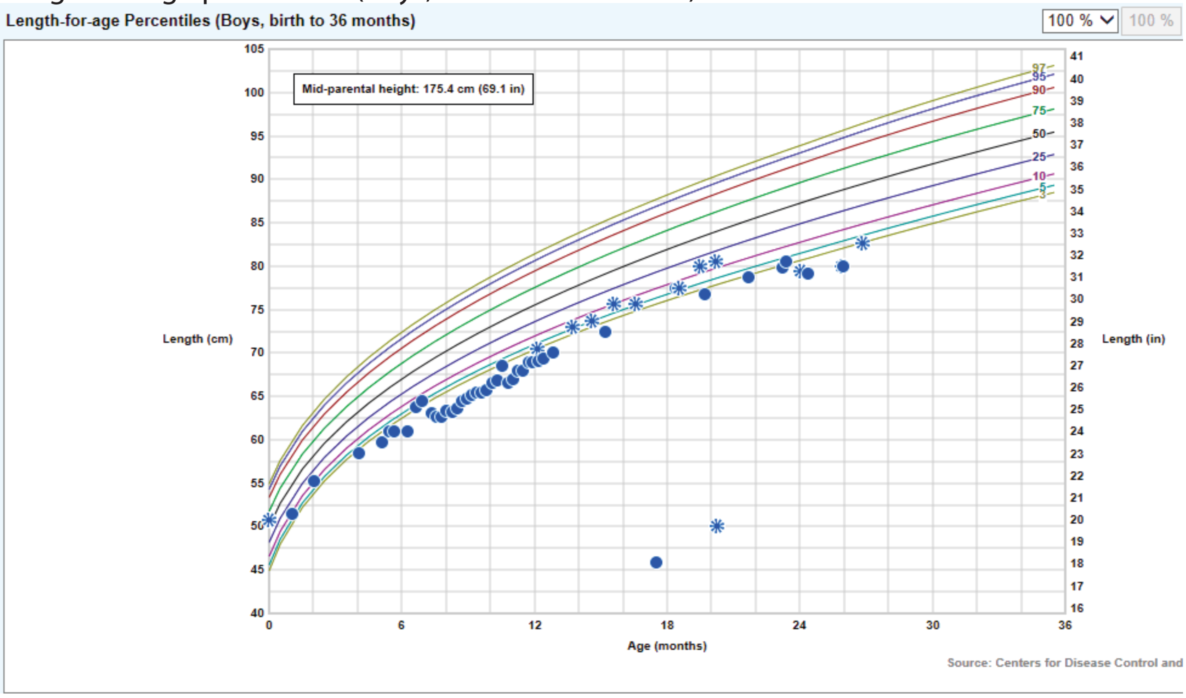

Weight for length percentiles (Boys, birth to 36 months)

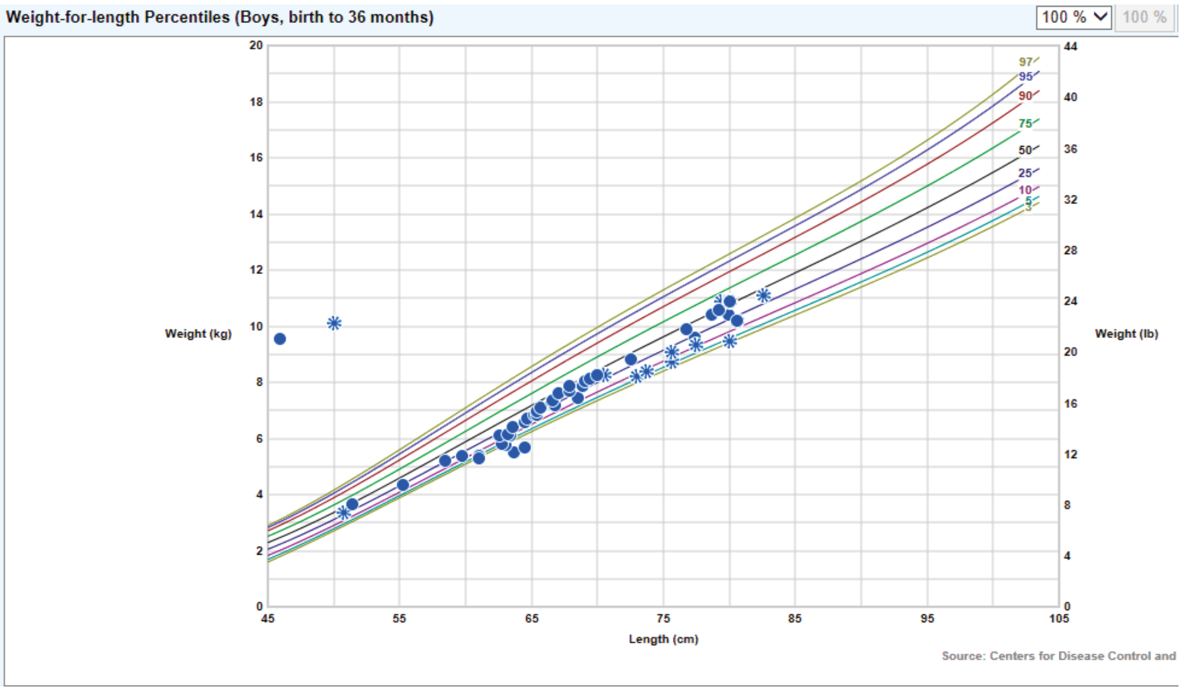

Supplemental Figure 1A. Growth charts for Patient 1

Weight for age percentiles (Girls, birth to 2 years)

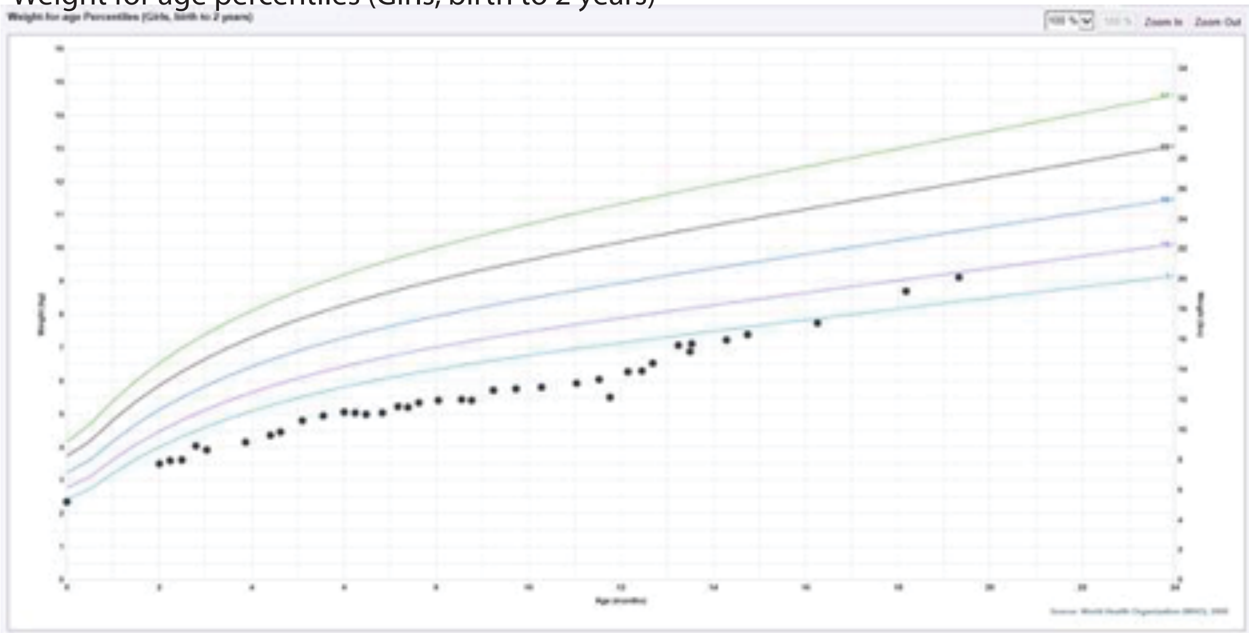

Length for age percentiles (Girls, birth to 2 years)

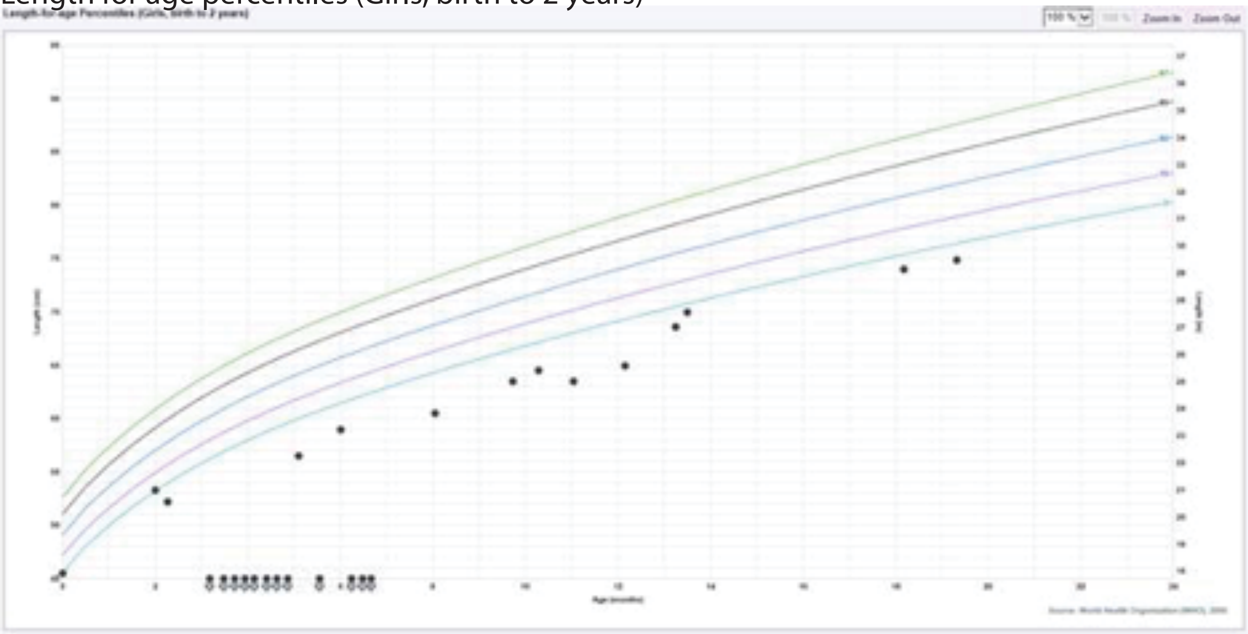

Weight for length percentiles (Girls, birth to 2 years)

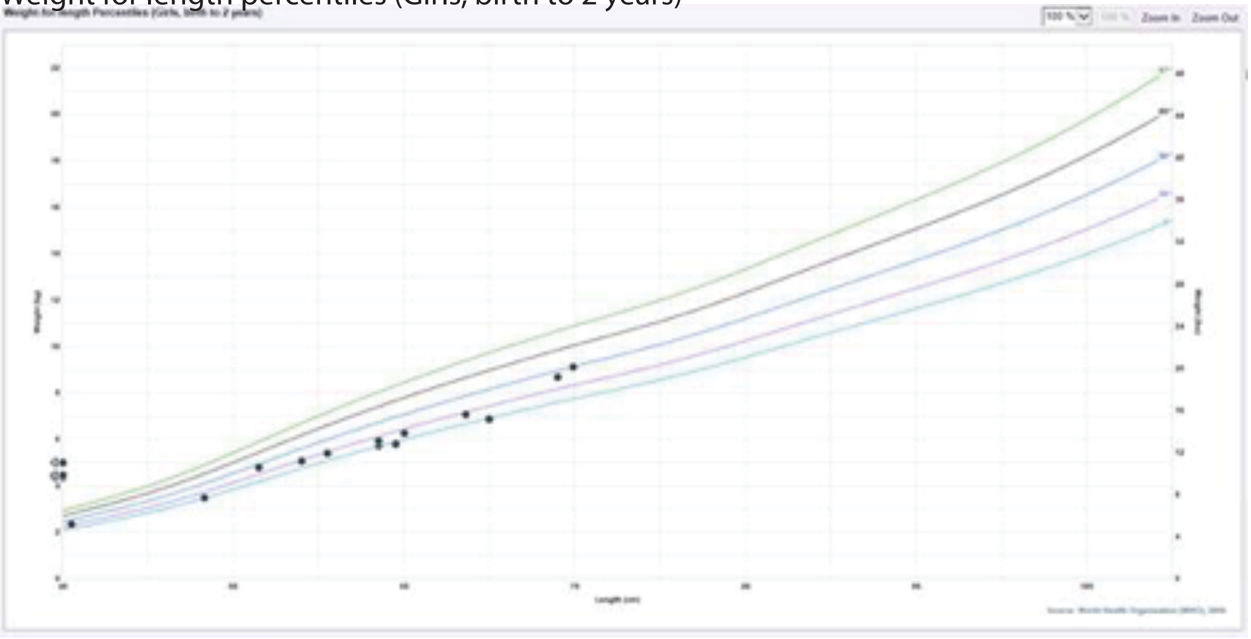

Supplemental Figure 1B. Growth charts for Patient 2
